# Survival Outcomes in Oral Tongue Cancer: A Mono-Institutional Experience Focusing on Age

**DOI:** 10.3389/fonc.2021.616653

**Published:** 2021-04-12

**Authors:** Mohssen Ansarin, Rita De Berardinis, Federica Corso, Gioacchino Giugliano, Roberto Bruschini, Luigi De Benedetto, Stefano Zorzi, Fausto Maffini, Fabio Sovardi, Carolina Pigni, Donatella Scaglione, Daniela Alterio, Maria Cossu Rocca, Susanna Chiocca, Sara Gandini, Marta Tagliabue

**Affiliations:** ^1^ Division of Otolaryngology and Head and Neck Surgery, IEO, European Institute of Oncology IRCCS, Milan, Italy; ^2^ Department of Experimental Oncology, IEO, European Institute of Oncology IRCCS, Milan, Italy; ^3^ Department of Mathematics, DMAT, Politecnico di Milano, Milan, Italy; ^4^ Center for Analysis Decisions and Society, CADS, Human Technopole, Milan, Italy; ^5^ Division of Pathology, IEO, European Institute of Oncology IRCCS, Milan, Italy; ^6^ Department of Otorhinolaryngology, Policlinico San Matteo, IRCCS, Pavia, Italy; ^7^ Department of Otorhinolaryngology, ASST Ovest Milanese, Legnano, Italy; ^8^ Division of Data Manager, IEO, European Institute of Oncology IRCCS, Milan, Italy; ^9^ Division of Radiotherapy, IEO, European Institute of Oncology IRCCS, Milan, Italy; ^10^ Department of Medical Oncology, Urogenital and Head and Neck Tumors Medical Treatment, IEO, European Institute of Oncology IRCCS, Milan, Italy; ^11^ Department of Biomedical Sciences, University of Sassari, Sassari, Italy

**Keywords:** tongue cancer, age, prognosis, head neck cancer, T-N tract, overall survival

## Abstract

**Objective:**

The prognostic role of age among patients affected by Oral Tongue Squamous Cell Carcinoma (OTSCC) is a topic of debate. Recent cohort studies have found that patients diagnosed at 40 years of age or younger have a better prognosis. The aim of this cohort study was to clarify whether age is an independent prognostic factor and discuss heterogeneity of outcomes by stage and treatments in different age groups.

**Methods:**

We performed a study on 577 consecutive patients affected by primary tongue cancer and treated with surgery and adjuvant therapy according to stage, at European Institute of Oncology, IRCCS. Patients with age at diagnosis below 40 years totaled 109 (19%). Overall survival (OS), disease-free survival (DFS), tongue specific free survival (TSFS) and cause-specific survival (CSS) were compared by age groups. Multivariate Cox proportional hazards models were used to assess the independent role of age.

**Results:**

The median follow-up time was 5.01 years (range 0–18.68) years with follow-up recorded up to February 2020. After adjustment for all the significant confounding and prognostic factors, age remained independently associated with OS and DSF (respectively, p = 0.002 and p = 0.02). In CSS and TSFS curves, the role of age seems less evident (respectively, p = 0.14 and p = 0.0.37). In the advanced stage sub-group (stages III–IV), age was significantly associated with OS and CSS with almost double increased risk of dying (OS) and dying from tongue cancer (CSS) in elderly compared to younger groups (OS: HR = 2.16 95%, CI: 1.33–3.51, p= 0.001; CSS: HR = 1.76 95%, CI: 1.03–3.01, p = 0.02, respectively). In our study, young patients were more likely to be treated with intensified therapies (glossectomies types III–V and adjuvant radio-chemotherapy). Age was found as a prognostic factor, independently of other significant factors and treatment. Also the T–N tract involved by disease and neutrophil-to-lymphocyte ratio ≥3 were independent prognostic factors.

**Conclusions:**

Young age at diagnosis is associated with a better overall survival. Fewer younger people than older people died from tongue cancer in advanced stages.

## Introduction

One of the hot topics in the field of head and neck tumors concerns the increased incidence of oral tongue squamous cell carcinoma (OTSCC) in young people (1, 2). The reason for these new epidemiological data is still undefined. Above all, etiopathogenesis and prognosis remain unclear when compared to the traditional population affected by oral tongue cancer, which is generally composed of people of over fifty years old with known risk factors such as heavy smoking and alcohol habits (3, 4).

The incidence of mobile tongue cancer in young patients is reported as increasing worldwide, especially in the last three decades (5–8). In particular, the analysis carried out by the National Cancer Institute Surveillance, Epidemiology and End Results Program (SEER) in 2011 on North American data shows that the overall incidence of OTSCC remained stable from 1975 to 2007 but increased in women and more specifically in the sub-population of young white women (9). Many conflicting groups have been published on the etiology, natural history, and prognosis of OTSCC in young adults. As early as 1970, tongue cancer in young people was supposed to be a distinct clinical entity that needed to be treated more aggressively than that in older adults (10). In 2011, Patel et al. also hypothesized a possible hormonal influence as the cause of these tumors (9). In this scenario, also chronic mucosal trauma is considered a possible cause for OTSS in young patients (11).

It is well known that the onset of OTSCC is related to smoking and alcohol abuse and in some countries with the habit of chewing betel leaves (12, 13). In addition, some authors have reported that gender distribution is different depending on the age of onset of oral cancer (12, 14, 15). While in the elderly, males account for over 70% of cases, this percentage drops to 50–65% under 45 years of age. This difference is consolidated if we consider that most of the young, non-smoking and non-drinking patients are reported to be women (12, 14, 15).

Currently, the primary treatment strategy for OTSCC is upfront surgery followed, in advanced stages, by radiotherapy or radio-chemotherapy based on final histopathological findings, according to international guidelines (16, 17).

Locoregional control of OTSCC has improved in recent decades; the reason could be more aggressive surgical resections supported by modern reconstructive methods with free flaps and advances in adjuvant treatment (18–21).

However, despite improved locoregional control, survival rates have remained stable or slightly improved over the past two decades with 5-year overall survival (OS) of approximately 60% for all stages and 33–54% in patients with locally advanced disease (16, 22).

Other treatment strategies such as neoadjuvant chemotherapy followed by surgery or upfront radio-chemotherapy have been employed for OTSCC without any significant improvement in survival (22–24).

In young patients, the prognosis of OTSCC is still controversial. Several early reports on squamous cell carcinoma (SCC) concluded that the disease was more aggressive and the prognosis lower in young adults compared to older patients (10, 25–27). However, recent studies have not found any significant differences in OS between different age groups (2, 28–32). At the same time, several other investigators claim that younger OTSCC patients have better survival compared to elderly patients (6, 8, 29, 33, 34). Conversely, the Memorial Sloan-Kettering Cancer Center reported that younger OTSCC patients had a higher rate of locoregional recurrence, with no significant difference in survival between young and old groups (35).

Many studies reported no difference in biological behavior among young and elderly patients with OTSCC (36–41). In particular, the latest next-generation sequencing techniques indicated that genomic profiles and mutations in the guide genes were very similar among young and elderly OTSCC patients, with similar mechanisms of tumorigenesis (36–41). Moreover, gene-specific mutation and copy number alteration frequencies were the same between young and old OTSCC patients in two independent cohorts (40).

Different results on prognosis and etiology may be attributed both to the small size of the patient cohorts and the different cut-off to define “young” age. Furthermore, there was a considerable heterogeneity both between and also within samples (*i.e.*, matched/unmatched, early/advanced tumor stage). Finally, they did not specify whether or not studies were adjusted for factors such as treatment modality, stage of the disease, presence and absence of metastatic lymph nodes, and percentage of patients between the two age groups. With these premises, the fundamental question about the role of age in cancer outcome of oral tongue cancer remains unanswered.

The aim of the study was to investigate the prognostic role of age and its influence on OTSCC cancer relapse and survival. We collected information on clinical and demographic characteristics of a retrospective cohort of OTSCC patients treated with homogenous modality in our institution, to define which factors could mostly influence survival outcomes.

## Material and Methods

Between January 2000 and December 2018, a total of 891 patients with oral cavity cancer underwent surgery at the Division of Otolaryngology and Head and Neck surgery of the European Institute of Oncology, IRCCS (IEO). The inclusion criteria were: patients with a histologically confirmed primary diagnosis of OTSCC and primary surgical treatment received at IEO. Among these, 173/891 patients were excluded because of previous surgery and/or excisional biopsy which were considered as a complete surgical therapy and not as a mere diagnostic procedure; 105/891 patients were excluded due to histology other than SCC, and 13/891 were excluded because the tumor origin was attributable to the base of the tongue. At the end of the selection, 577 patients with primary diagnosis of OTSCC and primary surgical treatment received at IEO were included in the study cohort.

The current study was approved by the IEO Ethics Committee (cod. IEO 225).

Staging referred to the TNM classification in accordance with the 7^th^ edition American Joint Committee on Cancer system, and all cases were re-classified according to the 8^th^ edition (42, 43). We reported the clinical stages according the 7^th^ TNM editions and the pathological stages in both 7^th^ and 8^th^ TNM editions for completeness of results.

For the study of the paper outcomes, we used the new staging system (8^th^ TNM edition) because it is the prognostic TNM currently used.

Preoperative diagnoses were assessed by a simple biopsy of the lesion, while magnetic resonance imaging or a computed tomography scan was performed for local disease study. Positron emission tomography/computedtomography or total body computed tomography was used for the pre-operative patients’ systemic evaluation.

The retrospective information extracted from the electronic medical records included:

- anthropometric and demographic patient characteristics: height, weight, age, gender, pre-operative neutrophil-to-lymphocyte ratio (NLR);
- epidemiologic data: smoking history, family history for tongue cancer and other cancers, alcoholic habits;
- histopathologic features and staging: surgical margins, clinical and pathological TNM 7^th^ and 8^th^ editions, tumor grading, the status of T–N tract (44, 45);
- performed treatment: type of surgery, glossectomies I to V according to the Ansarin et al. classification (from transoral to total glossectomy) (46), adjuvant treatment strategies (radiotherapy and/or chemotherapy)
- follow-up information: type of recurrence (local/locoregional) and/or distant metastases, secondary primary and the patients’ status at the last follow-up.

The definition of pack–years of cigarette smoking was used for the evaluation of tobacco consumption (47). Smoking and alcohol status at diagnosis were collected to classify patients as current, former, and never-smoking/drinking.

About the “neck lymph node” status we considered clinical (c)/pathological (p) Nx, N0, N+ as distinct variables. All patients’ follow-up were collected and updated to assess their status at the last clinical evaluation and the last contact. Patient deaths and their possible causes were assessed using the Italian national death register.

The NLR cutoff of < or ≥3 was determined based on prior publications (48–51).

All the data were collected in a well-designed database according to good clinical practice guidelines.

In previous studies of OTSCC the most frequent used age cutoff to define young adulthood was 40; moreover, it is reported that the role of risk factors, as smoke and alcohol, start to be significant after the age of 40 (1, 52–54). Thus, the main analysis was carried out considering 40 years of age as the cutoff of interest.

### Treatment Modality

All patients received the standard surgical treatment according to the IEO protocol for tongue cancer (46). The clinical early stages (clinical stags I and II) underwent trans-oral glossectomies (type I or II) followed by delayed (within 30 days) neck dissection (I–IV levels) in cases of deep of infiltration (DOI)>3 mm in the tongue. The “wait and see” policy was chosen where DOI was less than 3 mm.

The variable “neck dissection” (**Table 1**) refers to neck dissections performed *en bloc* with the tongue cancer (glossectomies types III to V) or as prophylactic neck dissection after 4 weeks from trans-oral glossectomies (types I or II) for tumor DOI.

**Table 1:** Patients’, tumor and treatments characteristics according to age.

		N (%):	Age ≤ 40 (%)	Age > 40 (%)	P-value
			N = 109	N = 468	
Gender	F	230 (36.57)	43 (39.45)	187 (39.96)	1
	M	347 (60.14)	66 (60.55)	281 (60.04)	
BMI	<24.9	301 (52.17)	57 (52.29)	244 (52.14)	0.13
	25.0–29.9	199 (34.49)	29 (26.61)	170 (36.32)	
	≥30	86 (14.9)	20 (18.35)	66 (14.1)	
	Unknown	11 (1.91)	3 (2.75)	8 (1.71)	
Smoking	Never	208 (36.05)	50 (45.87)	158 (33.76)	0.01
	Current/Former	362 (62.74)	56 (51.38)	306 (65.38)	
	Unknown	7 (1.21)	3 (2.75)	4 (0.85)	
Smoking pack/year	<20	326 (56.5)	89 (81.65)	237 (50.64)	<0.001
	≥20	232 (40.21)	14 (12.84)	218 (46.58)	
Alcohol	Never	288 (49.91)	79 (72.48)	209 (44.66)	<0.001
	Current/Former	280 (48.53)	27 (24.77)	253 (54.06)	
	Unknown	9 (1.56)	3 (2.75)	6 (1.28)	
Family history for tongue squamous cell carcinoma	No	546 (94.63)	101 (92.66)	445 (95.09)	0.69
	Yes	11 (1.91)	1 (0.92)	10 (2.14)	
	Unknown	20 (3.47)	7 (6.42)	13 (2.78)	
Family history for other squamous cell carcinomas	No	311 (53.9)	76 (69.72)	235 (50.21)	<0.001
	Yes	247 (42.81)	27 (24.77)	220 (47.01)	
	Unknown	19 (3.29)	6 (5.5)	13 (2.78)	
Grading	G1	104 (18.02)	19 (17.43)	85 (18.16)	0.62
	G2	267 (46.27)	48 (44.04)	219 (46.79)	
	G3	186 (32.24)	40 (36.7)	146 (31.2)	
	Unknown	20 (3.47)	2 (1.83)	18 (3.85)	
Neutrophil to Lymphocyte Ratio (NLR)	<3	378 (65.51)	86 (78.9)	292 (62.39)	0.006
	≥3	178 (30.85)	21 (19.27)	149 (31.84)	
Clinical Tumor (VII ed)	T1	186 (32.24)	32 (31.19)	152 (32.48)	0.96
	T2	173 (29.98)	33 (30.28)	140 (29.91)	
	T3–T4	218(37.78)	42 (38.53)	176 (37.61)	
Clinical lymph nodes (VII ed)	N0	367 (63.6)	63 (57.8)	304 (64.96)	0.19
	N+	210 (36.4)	46 (42.2)	164 (35.04)	
Pathological Tumor (VII ed.)	T1	186 (32.24)	56 (51.38)	130 (27.78)	0.29
	T2	104 (18.02)	28 (25.69)	76 (16.24)	
	T3–T4	248 (42.98)	58 (53.21)	190 (40.6)	
Pathological Tumor (VIII ed.)	T1	142 (24.61)	24 (22.02)	118 (25.21)	0.63
	T2	136 (23.57)	29 (26.61)	107 (22.86)	
	T3–T4	299 (51.82)	56 (51.38)	243 (51.92)	
Patological lymph nodes N (VII ed.)	N0	158 (27.38)	33 (30.28)	125 (26.71)	0.05
	N+	243 (42.11)	53 (48.62)	190 (40.6)	
	NX	176 (30.5)	23 (21.1)	153 (32.69)	
Patological lymph nodes (VIII ed.)	N0	158 (27.38)	33 (30.28)	125 (26.71)	0.05
	N+	243 (42.11)	53 (48.62)	190 (40.6)	
	NX	176 (30.5)	23 (21.1)	153 (32.69)	
Stage (VII ed.)	I	180 (31.2)	33 (30.28)	147 (31.41)	0.55
	II	70 (12.13)	12 (11.01)	58 (12.39)	
	III	36 (6.24)	10 (9.17)	26 (5.56)	
	IV	291 (50.43)	54 (49.54)	237 (50.64)	
Stage (VIII ed.)	I	130 (22.53)	19 (17.43)	111 (23.72)	0.33
	II	94 (16.29)	21 (19.27)	73 (15.6)	
	III	176 (30.5)	38 (34.86)	138 (29.49)	
	IV	177 (30.68)	31 (28.44)	146 (31.2)	
Lymphovascular invasion	No	548 (94.97)	104 (95.41)	444 (94.87)	1
	Yes	29 (5.03)	5 (4.59)	24 (5.13)	
Perineural infiltration	No	491 (85.1)	90 (82.57)	401 (85.68)	0.50
	Yes	86 (14.9)	19 (17.43)	67 (14.32)	
Tongue Intrinsic muscle infiltration	No	79 (13.69)	12 (11.01)	67 (14.32)	0.45
	Yes	498 (86.31)	97 (88.99)	401 (85.68)	
Tongue Extrinsic muscle infiltration	No	303 (52.51)	56 (51.38)	247 (52.78)	0.86
	Yes	273 (47.31)	53 (48.62)	220 (47.01)	
	Unknown	1 (0.17)	0 (0)	1 (0.21)	
T–N tract status	Free from disease	261 (45.23)	59 (54.13)	202 (43.16)	0.02
	Involved by disease	68 (11.79)	16 (14.68)	52 (11.11)	
	Not removed	248 (42.98)	34 (31.19)	214 (45.73)	
Extracapsular extension	No	461 (79.9)	89 (81.65)	372 (79.49)	0.70
	Yes	116 (20.1)	20 (18.35)	96 (20.51)	
Tumor Side in the tongue	Right	275 (47.66)	52 (47.71)	223 (47.65)	0.60
	Left	284 (49.22)	54 (49.54)	230 (49.15)	
	Bilateral	11 (1.91)	3 (2.75)	8 (1.71)	
	Median	7 (1.21)	0 (0)	7 (1.5)	
Neck dissection	No	176 (30.5)	23 (21.1)	153 (32.69)	0.02
	Yes	401 (69.5)	86 (78.9)	315 (67.31)	
Surgery on Tumor	Glossectomies I-II (transoral)	245 (42.46)	34 (31.19)	211 (45.08)	0.01
	Glossectomies III–V (Compartmental)	332 (57.53)	75 (68.80)	257 (54.91)	
Margins	Free	490 (84.92)	98 (89.91)	392 (83.76)	0.42
	Macroscopic involvement	14 (2.43)	1 (0.92)	13 (2.78)	
	Close	72 (12.48)	10 (9.17)	62 (13.25)	
	Unknown	1 (0.17)	0 (0)	1 (0.21)	
Radiotherapy	No	346 (59.97)	58 (53.21)	288 (61.54)	0.12
	Yes	231 (40.03)	51 (46.79)	180 (38.46)	
Adjuvant radio chemotherapy	No	478 (82.84)	80 (73.39)	398 (85.04)	0.005
	Yes	99 (17.16)	29 (26.61)	70 (14.96)	
Median follow-up (years)		5.01 (0–18.68)	4.22 (0–18.14)	2.63 (0–18.68)	
Overall Survival 5 years		0.65	0.73	0.62	
Disease Free Survival 5 years		0.54	0.60	0.51	
Cause Specific Survival 5 years		0.70	0.75	0.68	
Tongue Specific Free Survival 5 years		0.60	0.70	0.67	

Tumor clinically staged as intermediate and advanced (III and IV) were treated with glossectomies types III–to V *en bloc* neck dissection removing the T–N tract in pull-through or with trans-mandibular approaches (compartmental tongue surgery). Adjuvant treatment, such as radiotherapy or radio-chemotherapy, was recommended based on definitive histopathological findings.

### Definition of Endpoints

Local recurrence was defined as recurrence in the original tumor bed with the same histopathologic features of the primary tumor in the first three years after treatment. Regional recurrence was described as a metastatic disease in the head and neck region. Distant recurrence was defined as the presence of metastatic disease in all other locations. Any recurrence was reported as any local, regional, or distant metastasis, whichever occurred first. Disease-free survival (DFS) was defined as the time from surgery until any kind of tumor recurrence, including the occurrence of a second primary tumor or death from any cause, or the last contact date if alive with no recurrence. The last contact date was considered the last follow-up visit performed with the patient or the last telephone call ascertaining the patient’s state of health.

We considered the second tumor as an event of interest if it appeared after three years of treatment in the oral cavity or in other districts at any time after treatment (55).

Overall survival (OS) was defined as the time from surgery until the date of death from any cause, or the last contact date if alive (56). Patients’ deaths were assessed using the Italian national death registers.

The following tumor-specific clinical outcomes were also evaluated: Cause-specific survival (CSS) defined as the time from surgery until the date of death for tongue cancer. In case of no death for tongue cancer the observation was censored at the last follow-up visit or the date of death for other causes. Tongue specific free survival (TSFS) included the period after a successful treatment during which there were no signs and symptoms of the disease that was treated (tongue cancer) (56–59). The events considered for the TSFS were: local, locoregional recurrence and metastases only for tongue cancer. In case of no events or death for tongue cancer, the observation was censored at the last follow-up visit or the date of death for other causes.

### Statistical Analysis

Patient clinical-pathological and tumor characteristics were expressed as relative frequencies and percentages according to age. We choose 40 years old as a cutoff point for age in compliance with previous published studies that evaluated age in head and neck cancer (1, 10, 60–62). We conducted also a sensitivity analysis looking for the best cutoff point in our group for each survival outcome, and we investigated the role of age also as a continuous variable.

Univariate models were performed to evaluate the association of age and other prognostic factors (*e.g.*, smoking pack/year, T–N tract, surgery and stage of 8^th^ TNM edition) with clinical outcomes (DFS, OS, CSS and TSFS). Differences between survival curves were investigated with Log-rank tests and estimated using the Kaplan–Meier method. We assessed the independent prognostic role of age for each outcome with multivariate Cox Proportional Hazard models adjusted for all significant prognostic factors. Hazard ratio (HR) with 95% confidence intervals (CIs) from multivariate Cox proportional hazard models were reported. Sub-group analyses were conducted to investigate whether stage (8^th^ TNM edition) and surgery were associated with any cancer event as local recurrence, secondary primary (DFS) or death of any cause (OS) and recurrence related only to tongue cancer (TSFS) or death of tongue cancer (CSS) depending on age (56–59). We used a Chi-squared test to assess the association of age with frequencies of patients diagnosed recently (between 2010 and 2020), to investigate the influence of time of diagnosis and whether recent diagnoses were associated with sex. Finally, we evaluated whether the proportion of young patients (≤40 years old) was significantly associated with the type of surgery and stage (8^th^ TNM edition). All analyses were carried out with R 4.0 software (http://cran.r-project.org/), and *p*-values <0.05 were considered statistically significant.

## Results

### Patients’ Characteristics

Clinical-pathological and tumor characteristics of the study population are reported in **Table 1**. Among the young group (n = 109), the median age was 32 (range 27–37), while the median age was 61 (range 50–71) in the elderly group (n = 468).

We did not find any significant difference between the two groups (young and elderly) in terms of sex, BMI, family history for oral tongue tumor, tumor stage (I–IV, 7^th^ and 8^th^ TNM editions), pT (7^th^ and 8^th^ TNM editions), cT and cN according the 7^th^ TNM edition, the status of post-surgery margins, and radiotherapy (RT) as adjuvant treatment.

Among the elderly patients, we found a significantly higher number of current/former smokers (65.38 *vs* 51.38% for older *vs* younger respectively, p = 0.01) as well as patients with family history for other family members with SCC (47.01 *vs* 24.77% for older *vs* younger respectively, p = 0.001). Moreover, comparing the two groups, the youngest population was significantly more treated with type III–V glossectomies (54.9 *vs* 68.8% for older *vs* younger respectively; p =0.01) and with neck dissections (67.31 *vs* 78.9% for older *vs* younger, respectively; p = 0.02).

In the two age groups considered, the preoperative NLR ratio was highly significant for the population over >40 years old with a value equal or greater than 3 (p = 0.006).

Also, for the pathological state of the lymph nodes (pN 7^th^ and 8^th^ editions) and of the T–N tract status (free from disease, involved by disease, not removed), we find a difference between the two populations under examination (p = 0.05 and p = 0.02, respectively). Moreover, adjuvant radio-chemotherapy was administered more in young patients than in elderly (14.96 *vs* 26.61% for older *vs* younger respectively; p = 0.005).

In our sample, the proportion of young diagnoses (≤40 years old) does not seem to be significantly increased over the years: 55% were ≤40 years old between 2000 and 2010, while they were 45% between 2010 and 2020 (p = 0.63).

Furthermore, we also found that the proportion of female cancers was not different from the past: 53% female patients were found between 2000 and 2010, while they totaled 47% between 2010 and 2018 (p = 1).

### Events During Follow-Up

The median follow-up time was 5.01 years (range 0–18.68 years) with follow-up recorded up to February 2020.

Overall, we observed 149 (25.8%) locoregional recurrences, 38 (6.5%) distant metastases, and 15 (2.6%) locoregional with synchronous distant metastases as the first site of relapse.

Two hundred and forty-six patients (42.6%) died: 66% died from primary tumor (locoregional-distant recurrence), 10% died from a second different tumor, 12% from no cancer related causes, and 12% died from unknown causes.

Forty-six patients had a second primary tumor, of whom two were ≤40 years old.

**Figure 1:**
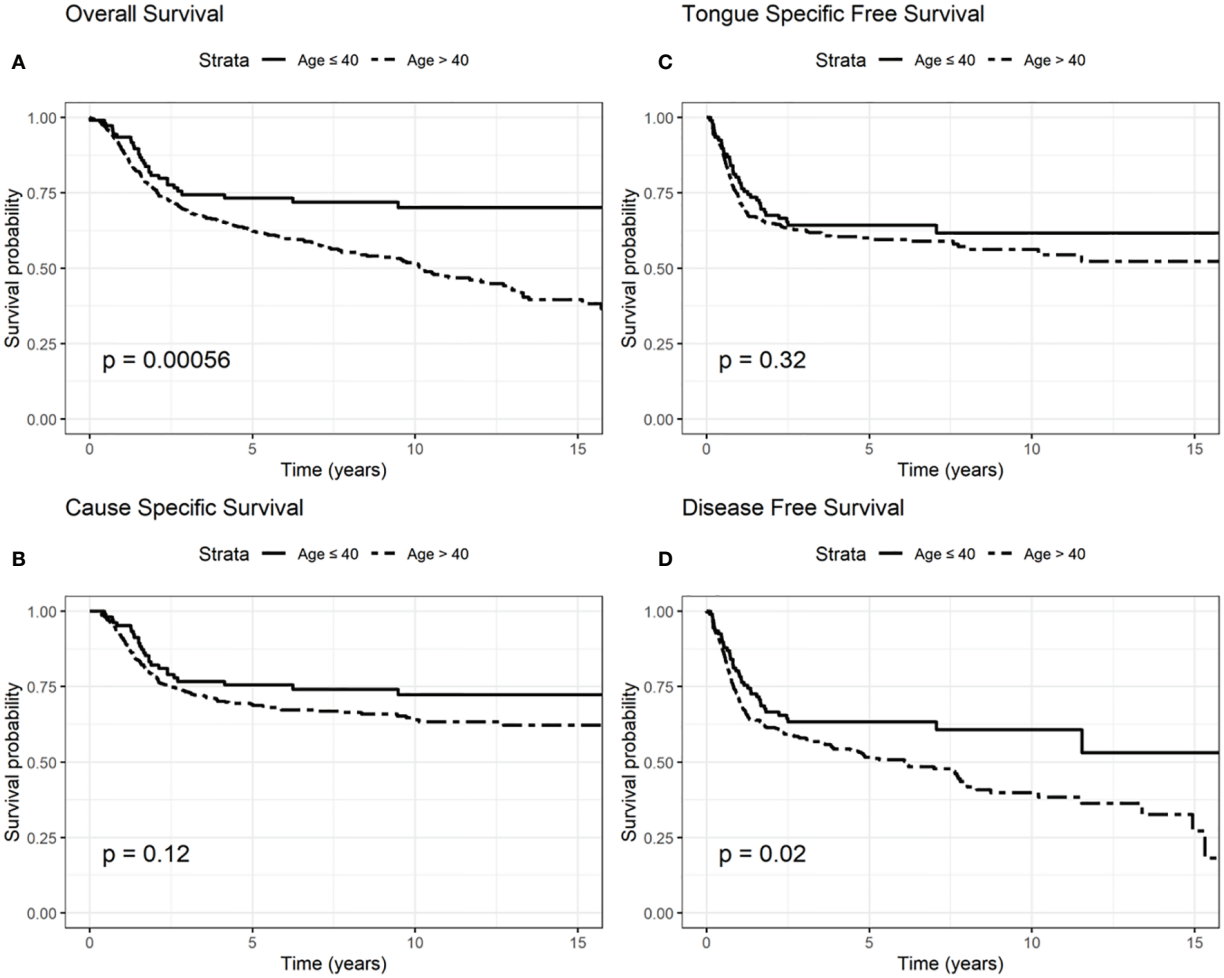
Survival probability: overall survival (OS) **(A)**, cause-specific survival (CSS) **(B)**, tongue specific free survival (TSFS) **(C)**, disease-free survival (DFS) **(D)** according to age.

Regarding OS, elderly 5-year survival was 62% compared to 73% among younger patients, while elderly 10-year survival was 51%, and 70% in younger patients (log rank test p = 0.0006, **Figure 1**).

CSS 5-year survival was 68 and 75% in elderly and younger patients, respectively; CSS 10-year survival was 63 and 72% among elderly and younger patients (log rank test p = 0.12, **Figure 1**).

TSFS at 5-year was 67% for elderly patients and 70% for younger patients; 10-year TSFS was 40% in case of the elderly compared to 50% in younger patients (log-rank test p = 0.32, **Figure 1**).

DFS at 5-year was 51% in elderly than 60% in younger patients; DFS at 10-year was 38 and 53% in elderly and younger patients, respectively (log-rank test p = 0.02, **Figure 1**).

**Figure 2:**
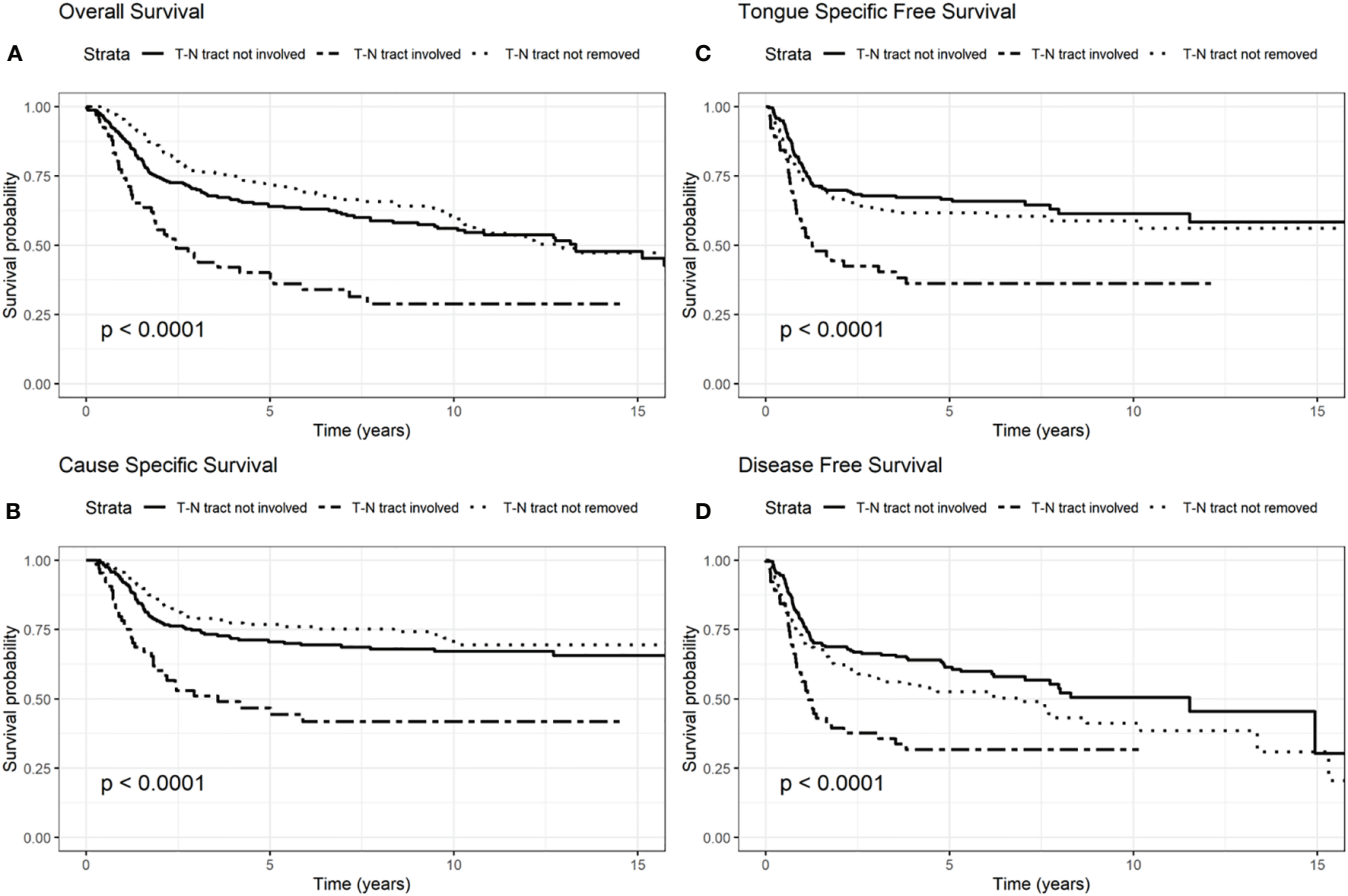
Survival probability: overall survival (OS) **(A)**, cause-specific survival (CSS) **(B)**, tongue specific free survival (TSFS) **(C)**, disease-free survival (DFS) **(D)** according to T–N tract status.

**Figure 3:**
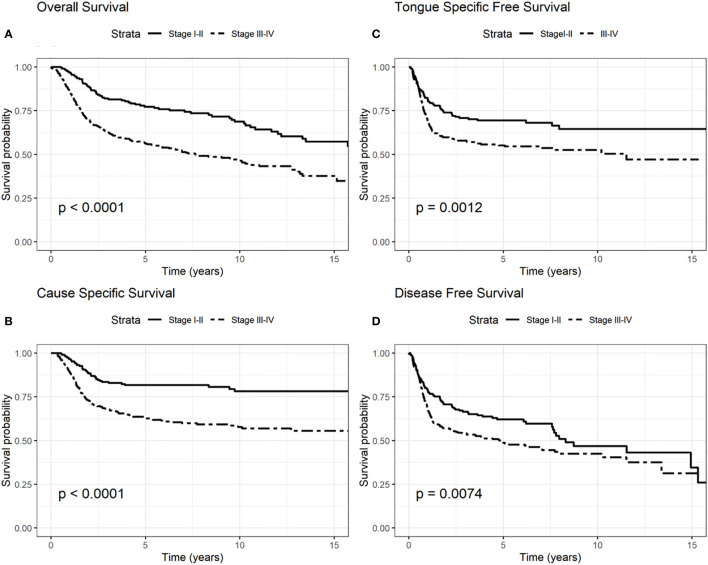
Survival probability: overall survival (OS) **(A)**, cause-specific survival (CSS) **(B)**, tongue specific free survival (TSFS) **(C)**, disease-free survival (DFS) **(D)** according to stages I–II and III–IV according to the 8^th^ edition.

We presented OS, CSS, TSFS, and DFS curves by age, T–N tract involvement and stage 8^th^ edition (**Figures 1**–**3**). Data on OS, CSS, TSFS, and DFS on smoking pack/year (p/y) with 20 p/y as cutoff point, type of intervention (III–V *vs* I, II glossectomies) and stage 8^th^ edition by univariate analysis in association with age (≤/>40) were not significant (data not shown).

In particular, we found that the elderly group was associated with worse overall survival (log-rank test p < 0.001) (**Figure 1**). These results were confirmed by the multivariate analysis, after adjusting for all significant prognostic factors, revealing an almost double risk of death in elderly patients (HR = 1.85 95%, CI: 1.24–2.76; p = 0.002) (**Table 2**). Age was still significantly associated with DFS, as a categorical variable, both in univariate (log-rank test p = 0.02) and multivariate Cox models with a 49% increased risk of relapse in elderly patients (HR = 1.49 95%, CI: 1.05–2.12; p = 0.02) (**Figure 1**, **Table 3**).

**Table 2:** Multivariate Cox model for OS.

Variable	Contrast	HR	Low.95	Up.95	P-value
Age	>40 *vs ≤*40	1.85	1.24	2.76	0.002
T–N tract status	Involved *vs* not involved by disease*	1.61	1.11	2.35	0.01
Stage (VIII ed.)	III *vs* I–II	1.73	1.20	2.49	0.003
	IV *vs* I–II	3.70	2.44	5.61	<0.001
Vascular Invasion	yes *vs* no	2.18	1.34	3.56	0.001
Radiotherapy	yes *vs* no	0.53	0.38	0.74	<0.001
NLR^	≥3 *vs <*3	1.47	1.12	1.92	0.004

*not involved by disease (T-N tract not removed for initial stage + removed but free from disease);

^NLR, Neutrophil to Lymphocyte ratio.

**Table 3:** Multivariate Cox model for DFS.

Variable	Contrast	HR	Low.95	Up.95	P-value
Age	>40 *vs ≤*40	1.49	1.05	2.12	0.02
T–N tract status	Involved *vs* not involved by disease*	1.48	1.00	2.18	0.04
pN (VIII ed.)	N+ *vs* N0	2.16	1.45	3.22	<0.001
	NX *vs* N0	2.55	1.72	3.78	<0.001
ECE	yes *vs* no	1.48	1.01	2.15	0.04
Vascular Invasion	yes *vs* no	1.58	0.96	2.61	0.07
NLR^	≥3 *vs <*3	1.31	1.00	1.72	0.04

*not involved by disease (T–N tract not removed for initial stage + removed but free from disease);

^NLR, Neutrophil to Lymphocyte ratio; ECE, extracapsular tumor extension.

Conversely, age was not significantly associated with CSS and TSFS (log-rank test p = 0.14 and p = 0.37 respectively) (**Tables 4**, **5**).

**Table 4:** Multivariate Cox model for CSS.

Variable	Contrast	HR	Low.95	Up.95	P-value
Age	>40 *vs ≤*40	1.37	0.89	2.12	0.14
T–N tract status	involved *vs* not involved by disease*	1.78	1.16	2.72	0.007
pN (VIII ed.)	N+ *vs* N0	3.27	2.05	5.24	<0.001
	NX *vs* N0	2.86	1.68	4.88	<0.001
pT (VIII ed.)	3–4 *vs* 1–2	1.88	1.25	2.83	0.002
NLR ^	≥3 *vs <*3	1.43	1.04	1.98	0.03
Adjuvant radio-chemotherapy	yes *vs* no	0.63	0.40	0.96	0.03

*not involved by disease (T–N tract not removed for initial stage + removed but free from disease).

^NLR, Neutrophil to Lymphocyte ratio.

**Table 5:** Multivariate Cox model for TSFS.

Variable	Contrast	HR	Low.95	Up.95	P-value
Age	>40 *vs ≤*40	1.17	0.82	1.68	0.37
T-N tract status	Involved *vs* not involved by disease*	1.66	1.13	2.45	0.009
Grading	G3–G2 *vs* G1	1.63	1.05	2.54	0.028
pT (VIII ed.)	III–IV *vs* I–II	1.55	1.08	2.24	0.017
pN (VIII ed.)	N+ *vs* N0	3.53	2.23	5.59	<0.001
	NX *vs* N0	3.97	2.75	7.67	<0.001

*not involved by disease (T–N tract not removed for initial stage + removed but free from disease).

The difference between heavy (<20 p/y) and non-heavy smokers (≥20 p/y) was found to be significantly associated with OS but only in the univariate analysis (log-rank p = 0.05).

The involvement of the T–N tract was found to be significantly associated with all the evaluated clinical outcomes (OS, CSS, DFS, TSFS with p < 0.001) (**Figure 2**, **Tables 2** **–** **5**). Similarly, patients with advanced stage IV appeared to have a worse prognosis (p < 0.001 for OS, CSS, and DFS) (**Figure 3**).

The multivariable Cox model for OS showed that age remained independently associated with death (p = 0.002), adjusting for T–N tract, stage, vascular invasion together with adjuvant RT and NLR (**Table 2**).

Age was independently associated with DFS (p = 0.02), adjusting for other significant prognostic factors such as the T–N tract involvement, pN, extra capsular tumor spread (ECE), vascular invasion, and NLR (**Table 3**).

On the other hand age was not found to be associated with CSS (p = 0.14) adjusting for T–N tract status, pN, pT NLR, and adjuvant radio-chemotherapy. The latter factors were found to be significantly associated with dying of tongue cancer (**Table 4**).

Finally, only T–N tract status, grading, pT and pN were independent prognostic factors associated with TSFS (**Table 5**).

In all the multivariate models in which the variable pN was found to be significant, we see how patients with pN+ and Nx always had a poorer prognosis compared to pN0 patients.

We also conducted stratified analyses to evaluate whether the association of age with recurrence (DFS and TSFS) and survival (OS and CSS) was different by stage (8^th^ edition) and treatment.

In the early stages (I–II), age was not found to be significantly associated with death (OS: HR = 1.56 95%, CI: 0.77–3.16, p = 0.21; CSS HR = 0.80 95% CI: 0.38–1.70, p = 0.57).

On the other hand, in multivariate analysis, for the advanced stages (III–IV, 8^th^ edition), age was found to be significantly associated with OS and CSS models, revealing a worse outcome for patients diagnosed at age >40 years (OS: HR = 2.16 95%, CI: 1.33–3.51, p= 0.001; CSS HR = 1.76 95%, CI: 1.03–3.01, p = 0.02) (**Tables 6**, **7**).

**Table 6:** Multivariate Cox model for OS, stages III–IV.

Variable	Contrast	HR	Low.95	Up.95	P-value
Age	>40 *vs ≤*40	2.16	1.33	3.51	0.001
T–N tract status	involved *vs* not involved by disease*	1.88	1.30	2.73	<0.001
Vascular Invasion	yes *vs* no	2.24	1.36	3.67	0.001
Radiotherapy	yes *vs* no	0.69	0.50	0.95	0.02
NLR^	≥3 *vs <*3	1.55	1.15	2.11	0.004

*not involved by disease (T–N tract not removed for initial stage + removed but free from disease).

^NLR, Neutrophil to Lymphocyte ratio.

**Table 7:** Multivariate Cox model for CSS, stages III–IV.

Variable	Contrast	HR	Low.95	Up.95	P-value
Age	>40 *vs ≤*40	1.76	1.03	3.01	0.02
T–N tract status	Involved *vs* not involved by disease*	1.97	1.29	3.00	0.004
pN (VIII ed.)	N+ *vs* N0	2.85	1.66	4.91	<0.001
	NX *vs* N0	3.31	1.55	7.06	0.001
NLR^	≥3 *vs <*3	1.51	1.04	2.17	0.02
Adjuvant radiochemotherapy	yes *vs* no	0.69	0.44	1.06	0.09

*not involved by disease (T–N tract not removed for initial stage + removed but free from disease); ^NLR, Neutrophil to Lymphocyte ratio.

Furthermore, we investigated the relationship between age, stage, and performed treatment (glossectomy types I–II *vs* IV–V): we found that for advanced stages (III–IV 8^th^ edition) there were no statistically significant different distributions of types of glossectomies between the two age groups considered (p = 0.07); instead in the initial stages (I–II 8^th^ edition), glossectomies III–V were significantly more frequent at age ≤40 (30%) than at age >40 (14%), (p = 0.02) (**Table S1**).

Then, we analyzed the role of age in multivariate analysis for patients treated with the same surgery stratified by stage.

In the initial stages I–II according to TNM 8^th^ edition for OS and CSS, in glossectomies types I–II we did not find any significant prognostic factor. In contrast, for type III–V glossectomies and early stages low number of events did not allow to estimate independent prognostic factors (data not shown).

For TSFS in type I–II glossectomies and stages I–II, the lymph nodal status (pN+ and pNx) remained significant (p < 0.001) (**Table S2**). Low number of events in early stage treated with type III–V glossectomies did not allow to identify independent prognostic factors associated with TSFS (data not shown).

Focusing on the DFS for early stages, in glossectomies types I–II we found that lymph node status was significantly associated with relapse: worse DFS was found in patients with laterocervical disease (p = 0.001) (**Table S3**). In early stage type III–V glossectomies, the NLR ratio remained significant (p = 0.03) (**Table S4**).

Concerning the advanced stages, for OS we had significance for the NLR ratio (p = 0.006) in type I–II glossectomies (data not shown). In type III–V glossectomies, age remained significant with the T–N tract, vascular invasion, post-operative radiotherapy treatment and NLR ratio (p = 0.001, p = 0.003, p = 0.006, p = 0.007and p = 0.04 respectively) (**Table S5**).

We did not find significant elements in CSS for type I–II glossectomies in advanced stages. In type III–V glossectomies for advanced stages CSS, we confirmed the variables: age, T–N tract, lymph node status and adjuvant radio-chemotherapy as independent risks factors for worse prognosis (p = 0.01, p = 0.01, p < 0.001, and p = 0.06, respectively) (**Table S6**).

In the TSFS model for advanced stages, in type I–II glossectomies, only the presence of pNx remained associated with prognosis (p = 0.05), while in type III–V glossectomies we found that the T–N tract and pN+ status were significantly associated with worse TSFS (p = 0.005 and < 0.001 respectively) (data not shown).

In DFS of advanced stages, NLR ratio was found to be significantly associated with relapse for type I–II glossectomies (p = 0.05) (data not shown); while for type III–V glossectomies age, T–N tract status, lymph node status, ECE and vascular invasion remained significantly associated with relapse (p = 0.04, p = 0.07, p = 0.001, p = 0.07 and p = 0.04 respectively) (**Table S7**).

Regarding the female sub-group, age appears to be significantly directly associated with prognosis also in multivariate analysis: patients older than 40 years have almost double increased risk of dying (OS) compared to younger groups (OS: HR = 2.02 95%, CI: 1.12–3.84, p = 0.01) (**Table S8**). In the male sub-group results were similar.

We also assessed several sensitivity analyses considering age as continuous variable and considering as cut-off point of age 45 and conclusion did not change (data not shown).

## Discussion

Our analyses confirmed better survival outcomes in young patients than in elderly patients.

We choose 40 as the cutoff age to distinguish young from older people in line with the study by Oliver et al. and others (1, 52, 53); moreover it is reported that the role of risk factors, such as smoke and alcohol, seems to be significant after the age of 40 (54).

In the multivariate Cox analysis for OS and CSS, the variable “age” remained highly significant for the OS model: young people were characterized by a better OS regardless of tumor stage, while CSS did not seem to be significantly correlated with age. However, CSS was related to pT, pN, T–N tract status, NLR, and adjuvant radio-chemotherapy.

Analyzing the advanced stage sub-group (stages III–IV), age seemed to be significant in OS and in CSS models with a double risk of dying and dying for tongue cancer in elderly compared to young people.

Focusing on the role of age in multivariate analysis for patients treated with the same surgery stratified by stage, “age” did not seem to play a role for glossectomies types I–II and stages I–II.

In advanced-stage and glossectomies type III–IV patients at age ≥40 showed to have approximately double risk of death compared to younger patients (CSS) and 50% increased risk of relapse.

Many published studies have shown no significant difference in prognosis between young and elderly patients (31, 34, 35, 63–65). However, Goldenberg D et al. affirmed that a better prognosis characterized young patients than older ones (29).

In a matched-pair analysis, Farquhar et al. described a greater recurrence incidence in young people <45 years old, but no differences in overall mortality in the two groups (30).

In 2019 Oliver at al. published a study with a high number of cases (under 40 years old) and revealed that young patients did not have a worse survival than elderly, as previously found in smaller cohorts: controlling for all confounding factors, patients under 40 had a significantly 9% higher 5‐year survival (77.1 *vs* 68.2%). In this work, Oliver et al. underline that age alone could not be a factor for treatment intensification beyond the standard of care (1).

Over the years, other authors have demonstrated that young patients have a worse prognosis and suggested that more aggressive approaches could improve locoregional control and OS (8, 10, 66, 67).

Conversely, Oliver et al. reported in a considerable sample that: “the intensification of treatment could be a source of significant increases in morbidity and cost of treatment, without any proven benefits at this time” (1).

Actually, also in our sample, young patients underwent more aggressive treatments as shown by a significant number of glossectomies types II–V (compartmental surgery), neck dissection, and adjuvant therapies.

Studying our data by type of surgery, the difference in surgical treatment seemed to be statistically evident in the initial stages (I–II) where young patients were treated more with aggressive surgery, but these differences did not remain significant in multivariate analysis. In this regard, as reported by Oliver et al., it will be interesting to further investigate whether the intensification of therapy, generally reserved for young patients, really leads to better disease control in young people than in the elderly (1).

Focusing on adjuvant therapy, regardless of age, multivariate analysis showed that radiotherapy remained significantly associated with OS and radio-chemotherapy with CSS; among surgical treatments were not significant, taking into account other factors as stage, pT, pN, and LNR.

Another aspect highlighted in our results was the role of the T–N tract. The T–N tract is the soft tissue between the primary tumor (T) and the neck lymph nodes (N) and it is composed by the sublingual and submandibular glands, mylohyoid muscle, lingual nerve, artery, and vein and all the stromal tissue, and lingual and sublingual lymph nodes of the compartment. This study confirmed that, independently of age, and other factors analyzed in multivariate analysis, the involvement of T–N tract was significant in all studied survival models (OS, DFS, TSFS, and CSS). Consequently, the status of the T–N tract played an important role in prognosis for patients with OTSCC regardless of age. In fact, patients with the T–N tract involved by disease had a 60% increased risk of dying (OS) and a 78% higher risk of dying from tongue cancer (CSS). These data got worse in advanced stages III–IV where OS and CSS worsen almost twice as much as in those who do not have the disease in the T-N tract.

The presence of cervical metastases at diagnosis and the T–N tract status were confirmed as important prognostic factors in all the survival models stratified by stage.

Focusing on lymph nodal status, patients with pN+ and pNx always showed a worse oncological outcome in multivariate analysis. The presence of neck metastases is directly related to the oncological stage and, consequently, to the prognosis.

Special consideration should be made for patients with pNx. In case of pNx, many published works showed that these patients have a worse OS and a higher frequency of local recurrence, especially compared to pN0 patients. In fact, pNx patients generally did not undergo the neck dissection because of clinical condition or because the “wait and see” protocol was applied. In this way, as reported by the literature, about 30% of these patients remained with undiagnosed neck micro-metastases and then a worse prognosis for local relapses (68–71). Our data confirm this evidence.

Regardless of treatment modality, the role of smoking is still debated among risk factors for the young. The prognosis of young patients with OTSCC is still undefined, and there exists a lack of clear definition of young and old patients in the published literature. Analyzing the OTSCC literature, as already reported, the concept of “young age” has been considered in varying ways: from below 30 going up to 45 years old. However, the majority of studies considered 40 years old as the major age below which patients were defined young (21, 22, 35, 52, 60, 61, 72–76). There is an agreement that in patients below 40 years old, there is too short smoking exposure to develop carcinogenic activity. Thus, chronic mucosal trauma, genetic and/or hormonal features could cause oral tongue cancer, but to date, no certain data have been proven (11, 40, 54, 77).

In our data smoking and alcohol did not influence the prognosis of the two groups in multivariate analysis.

In our sample the proportion of female cancers was not different from the past. On the contrary, in 2011 Patel et al. reported that OTSCC was increasing among young white individuals with age 18 to 44 years, particularly among white women (9). A recent study on Asiatic patients with tongue cancer described an increasing incidence particularly in young females. Younger females with tongue SCC had no significant history of smoking (78, 79). Regarding the prognosis of our study cohort, females older than 40 years have almost double increased risk of dying (OS) compared to younger.

Moreover, our study highlighted the important role of the NLR as independent prognostic factor. As well reported not only in head and neck cancers, a tumor-induced change of the immune system toward a pre-tumoral pattern and worse prognosis are related with high values of the NLR ratio (80). In our sample, an NLR value greater than three was associated with a worse prognosis in the curves for OS, CSS, and DFS, and the pejorative role was confirmed in the OS and CSS for advanced stage in patients treated with both type I–II and III–V glossectomies. These data also showed how the role of the immune system, in addition to the age and stage of the disease, could be a determining factor in cancer aggressiveness. However, further studies are needed to better understand it.

In this study, we presented survival data specifically related to tongue cancer highlighting how young patients died less specifically of tongue cancer (CSS and TSFS).

In the advanced stages, young age and NLR smaller than three were correlated with a better prognosis in terms of OS and CSS.

As reported by the literature, the elderly group shows worse outcomes, and this fact could be related to the associated comorbidities of older people regarding OS (6, 33). Instead, for CSS we may hypothesize a role of the immune system. Some studies attested an increase of LNR in the elderly: these data could favor tumor aggression with a worsening of the specific cancer outcomes (81).

Moreover, the independent and pejorative role of the pNx was well defined in multivariate analysis for CSS, DFS and TSFS. In our sample the pNx was mostly referred to the oldest group which generally had the less invasive surgery and to the application of the “wait and see” protocol with “personalized” surgical approaches also based on their health status.

Nevertheless, this work has several limitations, such as the limited number of young patients, the monocentric and retrospective nature of the data.

Despite this, to the best of our knowledge, this is one of the largest monocentric cohort studies, the first work to describe patients who underwent standard and replicable surgical treatments over a period of years, reporting comprehensive data of known risk factors, with a long and complete period of follow-up and in which the prognostic role of age, T–N tract, and NLR is clearly demonstrated.

## Conclusion

The peer-reviewed biomedical literature has shown that the role played by age in OTSCC prognosis is a matter of controversy. Our study revealed that young patients had a better prognosis and survived longer than elderly patients. Moreover, young people showed a slightly better recurrence-free survival, and they died less from tongue cancer than older patients, even in advanced tumor stages. In our sample, young patients seem more likely to be treated with intensified mode. Future studies, prospective and multicentric, will be needed to investigate the role of treatment intensification in young patients with OTSCC.

## Data Availability Statement

The original contributions presented in the study are included in the article/**Supplementary Material**. Further inquiries can be directed to the corresponding authors.

## Ethics Statement

The studies involving human participants were reviewed and approved by the European Institute of Oncology cometee (cod. IEO 225). Written informed consent to participate in this study was provided by the participants’ legal guardian/next of kin.

## Author Contributions

MA and MT conceptualized and drafted the study. RBe carried out literature review and revised the manuscript. SZ and LB revised the manuscript. RBr, GG, MC, FM, SC, and DA critically reviewed the manuscript for important intellectual content. DS, FS, and CP collected patients’ data. SG and FC realized statistical analysis. All authors contributed to the article and approved the submitted version.

## Funding

This work was partially supported by the Italian Ministry of Health with Ricerca Corrente and 5x1000 funds.

## Conflict of Interest

The authors declare that the research was conducted in the absence of any commercial or financial relationships that could be construed as a potential conflict of interest.
