# Supplementary material for: Survival Outcomes in Oral Tongue Cancer: A Mono-Institutional Experience Focusing on Age
Source: Front Oncol. 2021 Apr 12;11:616653. doi: 10.3389/fonc.2021.616653 (PMC8075362; doi:10.3389/fonc.2021.616653)
Supplement: Supplementary file 1 [file DataSheet_1.pdf]

Supplementary Tables

Table S1. Type of surgery (glossectomies I-II vs III-V) according to age (Age ≤ 40, > 40) and tumor Stage 8th TNM edition (initial I-II and advanced III, IV).

		Age ≤ 40	Age>40	P-value
Stage^ I-II	Glossectomies I-II<br>Glossectomies III-V	28 (70%)<br>12 (30%)	158 (86%)<br>26 (14%)	p=0.02
Stage^ III-IV	Glossectomies I-II<br>Glossectomies III-V	6 (9%)<br>63 (91%)	53 (19%)<br>231 (81%)	p=0.07
^ Stage according to 8th TNM ed				

Table S2. Multivariable Cox proportional hazard model for TSFS in glossectomies I-II stage I-II

Variable	Contrast	HR	Low.95	Up.95	P-value
Age	> 40 vs ≤ 40	0.77	0.40	1.51	0.46
Grading	G3-G2 vs G1	1.80	0.92	3.50	0.08
	N+ vs N0	56.61	5.69	562.42	<0.001
pN (VIII ed.)	NX vs N0	1.75	0.82	3.74	0.14

Table S3. Multivariable Cox proportional hazard model for DFS in glossectomies I-II stage I-II

Variable	Contrast	HR	Low.95	Up.95	P-value
Age	> 40 vs ≤ 40	1.07	0.58	1.99	0.81
pN (VIII ed.)	N+ vs N0	31.95	3.64	280.13	0.001
	NX vs N0	1.14	0.65	2.00	0.63

Table S4. Multivariable Cox proportional hazard model for DFS in glossectomies III-V stage I-II.

Variable	Contrast	HR	Low.95	Up.95	P-value
Age	> 40 vs ≤ 40	2.91	0.25	33.73	0.39
NLR^	≥ 3 vs < 3	13.55	1.22	149.48	0.03
^NLR = Neutrophil to Lymphocyte ratio					

Table S5. Multivariable Cox proportional hazard model for OS in glossectomies III-V stage III-IV

Variable	Contrast	HR	Low.95	Up.95	P-value
Age	>40 vs ≤ 40	2.23	1.35	3.69	0.001
TN tract status	Involved vs not involved by disease*	1.76	1.21	2.56	0.003
Vascular Invasion	yes vs no	2.06	1.22	3.47	0.006
Radiotherapy	yes vs no	0.61	0.43	0.87	0.007
NLR^	≥ 3 vs < 3	1.40	1.00	1.97	0.04

* not involved by disease (T-N tract not removed for initial stage + removed but free from disease); ^NLR = Neutrophil to Lymphocyte ratio

Table S6 Multivariable Cox proportional hazard model for CSS in glossectomies III-V stage III-IV.

Variable	Contrast	HR	Low.95	Up.95	P-value
Age	> 40 vs ≤ 40	1.95	1.12	3.40	0.01
TN tract status	involved vs not involved by disease *	1.73	1.13	2.64	0.01
pN(VIII ed.)	N+ vs N0	2.86	1.65	4.94	<0.001
NLR ^	≥ 3 vs < 3	1.37	0.92	2.02	0.11
Adjuvant radio-chemotherapy	yes vs no	0.65	0.42	1.02	0.06

* not involved by disease (T-N tract not removed for initial stage + removed but free from disease); ^NLR = Neutrophil to Lymphocyte ratio

Table S7. Multivariable Cox proportional hazard model for DFS in glossectomies III-V stage III-IV

Variable	Contrast	HR	Low.95	Up.95	P-value
Age	> 40 vs ≤ 40	1.57	1.00	2.45	0.04
TN tract	Involved vs not involved by disease*	1.43	0.96	2.12	0.07
pN (VIII ed.)	N+ vs N0	2.39	1.41	4.03	0.001
ECE^	yes vs no	1.44	0.96	2.14	0.07
Vascular Invasion	yes vs no	1.72	1.01	2.92	0.04

* not involved by disease (T-N tract not removed for initial stage + removed but free from disease); ^ECE = extracapsular tumor spread

Table S8. **Multivariable Cox proportional hazard model for OS in female subgroup.**

Variable	Contrast	HR	Low.95	Up.95	P-value
Age	>40 vs ≤ 40	2.07	1.12	3.84	0.01
T-N tract	Involved vs not involved by disease*	2.51	1.22	5.18	0.01
Stage (VIII ed.)	III-IV vs I-II	1.79	1.10	2.88	0.01
Radiotherapy	yes vs no	0.45	0.26	0.79	0.005
NLR^	≥ 3 vs < 3	2.21	1.44	3.39	<0.001

* not involved by disease (T-N tract not removed for initial stage + removed but free from disease); ^NLR = Neutrophil to Lymphocyte ratio
